# Challenges in Measuring In Vitro Activity of LNP-mRNA Therapeutics

**DOI:** 10.3390/ijms26178152

**Published:** 2025-08-22

**Authors:** Giuditta Guerrini, Diletta Scaccabarozzi, Dora Mehn, Ambra Sarracino, Sabrina Gioria, Luigi Calzolai

**Affiliations:** European Commission, Joint Research Centre (JRC), 21027 Ispra, Italy; giuditta.guerrini@ec.europa.eu (G.G.); diletta.scaccabarozzi@ec.europa.eu (D.S.); dora.mehn@ec.europa.eu (D.M.); ambra.sarracino@ec.europa.eu (A.S.); sabrina.gioria@ec.europa.eu (S.G.)

**Keywords:** LNP-mRNA, nanomedicine, drug delivery, therapeutics, analytical ultracentrifuge (AUC), vaccine, quality, RNA encapsulation, stability

## Abstract

MRNA-based therapeutics and vaccines represent a rapidly expanding frontier in biomedical innovation, with lipid nanoparticles (LNPs) serving as a clinically validated delivery platform. This study explores critical quality attributes of LNP-mRNA formulations, with a particular focus on in vitro biological activity, a key quality attribute of vaccine activity and batch-to-batch consistency. We discuss the importance of optimizing both LNP components and mRNA structure, highlighting recent advances in formulation strategies. Furthermore, we examine the influence of factors such as cell-line selection, experimental design, storage conditions, and targeted cellular delivery on transduction efficiency. Our findings underscore the need for standardized in vitro assays and process-integrated monitoring to support the scalable development and regulatory assessment of mRNA-based therapies.

## 1. Introduction

Gene therapies and vaccines represent the most-rapidly advancing areas within the life sciences, with RNA technologies being at the forefront of much of this innovation [1]. The future of precision healthcare is being revolutionized by RNA-based medicines, which are transforming treatment approaches across various diseases. Breakthroughs in synthetic biology, the design of RNA sequences, and novel delivery methods are expected to overcome existing hurdles and unlock the full therapeutic potential of RNA. With technological innovations and growing commercial uses driving growth, the global RNA therapeutics market is projected to exceed $15 billion by the end of the decade, according to analyst estimation [2]. Among the various RNA delivery systems, lipid nanoparticles (LNPs) have emerged as a clinically validated platform. They are used in applications ranging from gene therapies—such as Onpattro^®^—to vaccines, most notably those developed for the prevention of SARS-CoV-2 disease (COVID-19) [3,4]. LNPs are also under investigation in clinical trials for broader applications including CRISPR-Cas9 gene editing, protein replacement therapies, and cell therapies [5,6,7,8].

In a previous study, we developed a comprehensive characterization strategy for COVID-19 LNP vaccines, including their immunomodulatory properties, and intracellular fate [9]. We also emphasized the critical need to standardize development processes to address common pitfalls during quality assessment. LNPs are complex, tunable systems composed of several key components: ionizable cationic lipids, one or more neutral/structural lipids, PEGylated lipids, the RNA payload, and a buffering system. The ionizable lipid drives mRNA encapsulation during LNP formation and supports its endosomal escape upon cell entry. Structural lipids like DSPC and cholesterol provide particle stability and membrane rigidity, while PEGylated lipids contribute to colloidal stability and prolonged circulation in vivo [10,11]. Synthesis media and storage, although sometimes overlooked, play an essential role in promoting particle assembly, lipid-mRNA adduct formation and LNP stability maintenance [12].

Beyond composition, other variables significantly impact LNP performance. Factors such as particle shape, surface charge, and cell-specific properties—including membrane elasticity, receptor density, and endocytic capability—make the interaction at the nanoparticle–cell interface highly heterogeneous. As a result, optimal LNP size and uptake kinetics can differ significantly across cell types and formulations [13]. Because these physicochemical properties strongly influence both formulation stability and in vivo delivery efficiency, considerable efforts have been made to refine LNP design and mRNA formulation strategies [14,15,16,17].

Optimizing the mRNA cargo itself is equally critical. Enhancements to regulatory elements—including the 5′ cap, 3′ poly(A) tail, and untranslated regions (UTRs)—have been shown to improve both mRNA stability and translational efficiency by promoting interaction with host-cell translation machinery [18]. Additionally, codon optimization and increased guanine–cytosine (G–C) content can stabilize mRNA secondary structures, extending the molecule’s functional half-life [19]. Incorporating chemically modified nucleotides, such as 1-methyl-pseudouridine (m^1^Ψ) or 5-Methoxy-Uridine (MoU), helps to evade innate immune detection and supports high levels of protein expression [20].

Assessing the quality and batch-to-batch consistency of LNP-mRNA formulations across both developmental and manufacturing scales requires comprehensive comparability analyses. These should include LNP size distribution, RNA content, RNA encapsulation efficiency (EE), and most importantly, the biological activity of the final product. “In vitro” transfection assays, using appropriate cell models, serve as an essential tool to evaluate protein expression, while also supporting ethical considerations by minimizing animal use in line with 3Rs principles.

The European Medicines Agency (EMA) guidelines on the quality aspects of mRNA vaccines [21] suggest cell-based in vitro testing as an option for studying whether the mRNA is translated to the correct protein product, while the European Pharmacopeia (Ph. Eur.) general chapter on the quality attributes of mRNA vaccines emphasizes the need for biological activity assays of the therapeutic mRNA product. Such assays must be sensitive, quantitative, and reproducible, and should ideally assess functional protein expression in physiologically relevant cell models. Despite its importance, the development and harmonization of robust, reproducible in vitro potency assays remains a significant challenge [14]. Ongoing harmonization efforts aim to define acceptable assay platforms and validation criteria to ensure consistency, particularly for products entering the market through centralized regulatory procedures.

In addition to the intrinsic properties of the nanomedicine formulation, apolipoprotein E (ApoE) (a plasma protein involved in lipid transport and metabolism) has been found to play a crucial role in the cellular uptake of LNPs [22]. ApoE can adsorb onto the surface of LNPs once they enter the bloodstream, mediating their recognition and uptake by cells expressing LDL-receptor family members. This mechanism can significantly enhance transduction efficiency, especially in hepatocytes, which express high levels of ApoE receptors (ApoERs). The efficacy of ApoE-mediated uptake can vary across different cell lines due to differential expression of ApoERs, cellular endocytic capacity, and the local lipid environment. These findings highlight the importance of considering plasma protein interactions when designing LNP formulations, particularly in the context of targeted delivery and personalized medicine approaches [23].

In this study, we describe the implementation of process-integrated protocols for monitoring in vitro activity—a critical quality attribute in LNP-mRNA development. We demonstrate the impact of selecting an appropriate cell line for protein expression, and how storage conditions and LNP targeting influence the efficiency of cellular transduction. Our findings offer insights into strategies that can support the development of more robust, scalable, and effective RNA-based therapeutics.

## 2. Results

### 2.1. LNP-mRNA Synthesis

Four different LNP-mRNA samples, each carrying mRNA encoding the firefly luciferase protein (FLuc mRNA), were synthesized in house, as described in the Section 4. All formulations were prepared using a pre-mixed lipid solution (GenVoy-ILM™, Cytiva, Marlborough, MA, USA) composed of ionizable lipid, cholesterol, DSPC, and PEG-lipid in a standard molar ratio of 50:37.5:10:2.5. LNP-1 and LNP-2, representing different batches with identical lipid and mRNA composition, were synthesized via the classical microfluidic system, in which the lipid phase (dissolved in ethanol) was rapidly mixed with the FLuc mRNA in an acidic aqueous buffer. LNP-3 was generated by incubating the LNP-2 formulation with apolipoprotein E (ApoE) post-synthesis, whereas LNP-4 was produced by co-encapsulating the ApoE protein with the mRNA during the LNP synthesis process.

Table 1 summarizes the composition and key physicochemical properties of the four samples. LNP-1 and LNP-2 have particle sizes in the expected range (70–80 nm) with a low polydispersity index (PDI), consistent with well-formed nanoparticles. LNP-3, produced by incubating with ApoE post-synthesis, showed a slight increase in size. Notably, LNP-4 displayed a significant size increase, reaching up to ~130 nm. The yield of encapsulation (YE) exceeded 90% for LNP-1 and LNP-2. In contrast, LNP-4 had a markedly lower YE (~13%), suggesting that the presence of ApoE during the formulation may interfere with a proper LNP-mRNA self-assembly or result in mRNA distribution on the particle surface rather than within the core. Supporting this hypothesis, a strong fluorescence signal was also observed in the non-lysed LNP-4 sample, indicating a significant proportion (63%) of unencapsulated or surface-associated mRNA. As a reference, the amount of unencapsulated or surface-associated mRNA for LNP-1 and LNP-2 were 7% and 2%, respectively.

### 2.2. LNP-mRNA In Vitro Activity

#### Effects of LNP-mRNA Treatment on Different Cell Lines

The in vitro activity of LNP-mRNA formulations is primarily determined by two critical factors: the efficiency of cellular uptake and the successful expression of the encoded protein. As such, it serves as a reliable surrogate endpoint for assessing the quality and functional efficacy of LNP-mRNA products without the need for animal testing. This approach aligns with current regulatory interests, as both the European Pharmacopeia and the US Pharmacopeia [24] are actively considering in vitro activity assays as part of the quality-control and batch-release criteria for mRNA-based vaccines and therapeutics. In addition, the EMA suggests the use of cell-based functionality assays, developed to target a particular antigen, to confirm the LNP uptake, endosomal escape, and efficient mRNA translation [21]. These checks are foreseen both at the release of the finished product and to test shelf life.

**Figure 1:**
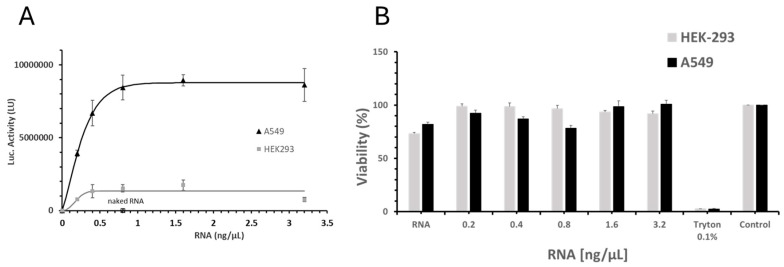
(**A**) Luciferase activity (LU) in HEK-293 (grey square) and A549 (black triangle) cells following treatment with LNP-1 at increasing mRNA doses (0.2, 0.4, 0.8, 1.6, and 3.2 ng/μL). Naked FLuc mRNA (naked RNA, empty triangle and empty square for HEK-293 and A549 respectively) and untreated cells (Ctl) were included as negative controls. Values represent the mean ± standard error of the mean for three independent luciferase activity assays. (**B**) Cell viability assessed by MTT assay in HEK-293 (grey) and A549 cells (black) treated with LNP-1 across a range of mRNA doses (0.2, 0.4, 0.8, 1.6, and 3.2 ng/μL). Naked FLuc mRNA (RNA), Triton-treated cells (positive control for cytotoxicity), and untreated cells (Ctl) were used as controls. Values represent the mean ± standard error of the mean for three independent luciferase activity assays.

To explore this further, we assessed the in vitro activity of the LNP-1 formulation in two commonly used cell lines—HEK-293 and A549—by quantifying the expression of firefly luciferase (Fluc) using a standard luminescence-based detection assay (Figure 1A). This comparison allowed us to evaluate how cell type influences LNP uptake and functional protein expression. The applied assay measures luciferase activity in the cell lysate, eliminating protein-excretion-capacity difference as a factor when comparing different cell lines.

HEK-293 cells (derived from human embryonic kidney cells) are widely used as a standard cell line for in vitro protein expression studies due to their high transfection efficiency with both liposomal and non-liposomal reagents [25]. These cells are considered to be robust, viable, and easy to culture, making them a reliable platform for transfection assays. In parallel, A549 cells (originating from human alveolar basal epithelial adenocarcinoma) are commonly used in both fundamental biological research and drug discovery, particularly in studies related to respiratory diseases and epithelial cell biology.

Figure 1A illustrates the luciferase expression profile following transfection with LNP-mRNA at increasing mRNA concentrations in both HEK-293 and A549 cells. Luciferase activity exhibited a dose–response relationship: it increased with mRNA concentrations up to 0.8 ng/μL (equivalent to 40 ng total mRNA per well), plateaued at 1.6 ng/μL, and showed a slight decline at the highest dose tested of 3.2 ng/μL. Cell viability (measured using the MTT assay, Figure 1B) for all tested LNP-mRNA concentrations was always above 85% of the control, thus indicating no apparent cytotoxic effects from the LNP-mRNA formulations.

Even if the dose–response profile was very similar for the two tested cell lines (with the luciferase activity plateauing at 1.6 ng/μL) the luciferase activity was significantly higher in A549 cells compared to HEK-293 across all tested mRNA concentrations. These findings highlight the critical influence of cell type on in vitro transfection outcomes. Differential protein expression following LNP-mRNA delivery can be attributed not only to inherent cellular differences (such as receptor expression, endosomal processing, and cellular translation and excretion capacity), but also to the specific characteristics of the LNP formulation, particularly surface functionalities [26,27] and the choice of ionizable lipid, which governs cellular uptake and endosomal escape efficiency [28]. The marked disparity in luciferase expression between the two cell lines upon transfection with the same LNP-1 formulation underscores the necessity of selecting an appropriate cellular model for in vitro potency assays.

**Figure 2:**
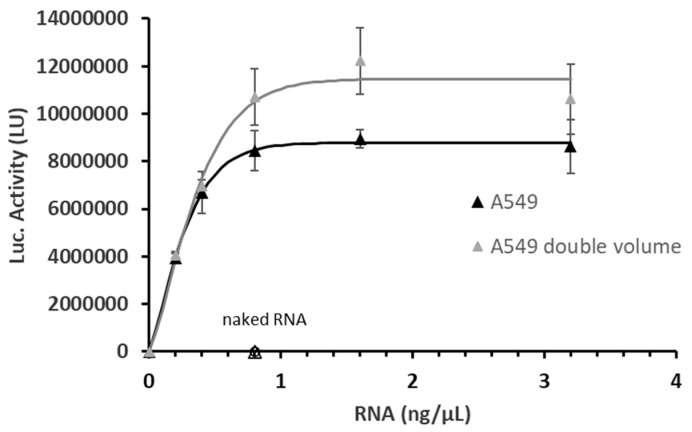
Luciferase activity (LU) in A549 cells treated with LNP-1 across a range of mRNA doses (0.2, 0.4, 0.8, 1.6, and 3.2 ng/μL), comparing two total volumes per well: 100 μL (grey triangles) and 50 μL (black triangles). Naked FLuc mRNA (naked RNA, empty triangle and empty circle for HEK-293 and A549 respectively) were included as negative control. Although the final mRNA concentrations were identical for each dose, the total mRNA mass differed due to the varying treatment volumes. Values represent the mean ± standard error of the mean for three independent luciferase activity assays.

We next evaluated the impact of the total volume of the medium (culture medium plus treatment solution) on luciferase activity. As is shown in Figure 2, luciferase output was not only dependent on cell number but was also strongly influenced by the total mass of mRNA delivered per well. Specifically, at a fixed mRNA concentration (e.g., 0.8 ng/μL), increasing the volume of the medium (and thus the total mRNA dose from 40 ng to 80 ng per well) resulted in a significant increase in luciferase activity.

### 2.3. Impact of Thermal Stress and Freeze–Thaw Cycles on LNP-mRNA Stability and Functionality

#### Efficacy of LNP-mRNA Formulations: Sensitivity to Temperature Stress

Temperature stress during the synthesis process and the storage of LNP-mRNA formulations can significantly impact their stability, quality, and translational efficacy.

To investigate this, A549 cells were treated with aliquots of the same LNP-2 formulation that had been subjected to various temperature conditions: storage at +4 °C, incubation at +37 °C or +85 °C for 15 min, and repeated freeze–thaw cycles at −80 °C, as detailed in the Section 4.

**Figure 3:**
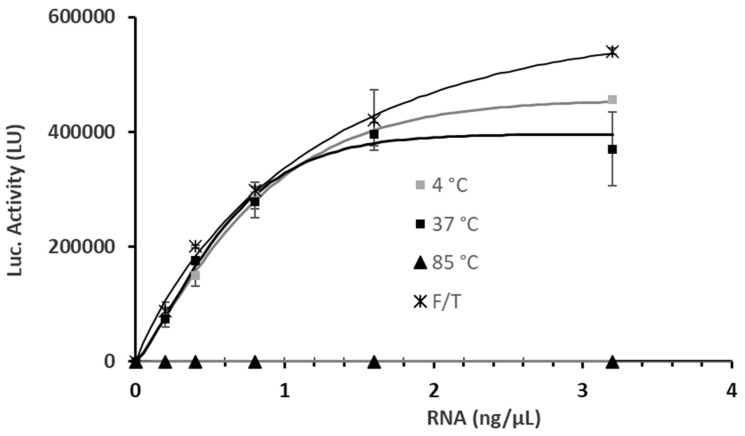
Luciferase activity (LU) in A549 cells treated for 24 h with LNP-2 formulations across a range of mRNA doses (0.2, 0.4, 0.8, 1.6, and 3.2 ng/μL), and subjected to different storage conditions: +4 °C (grey squares), +37 °C (black squares), +85 °C (black upward triangles), and repeated freeze–thaw cycles (black asterisks). Untreated cells (Ctl) were included as a negative control. Values represent the mean ± standard error of the mean for three independent luciferase activity assays.

Luciferase activity data (Figure 3) indicate that LNP-2 preserved its ability to protect the mRNA cargo and support protein expression following storage at +4 °C, which is consistent with previous reports demonstrating adequate stability at this temperature [18]. Furthermore, short-term exposure at +37 °C did not result in any significant loss of activity, suggesting that the structural integrity of the LNPs—and their encapsulated mRNA—was maintained under mild thermal stress.

Exposure to thermal stress can severely compromise the structural and functional integrity of LNP-mRNA formulations. In our study, incubating LNP-2 at +85 °C for 15 min led to a complete loss of luciferase expression in A549 cells, indicating total inactivation of the mRNA cargo (Figure 3). Analytical ultracentrifugation (AUC) confirmed extensive LNP disruption and mRNA release under these conditions. Once released from the protective lipid shell, mRNA becomes vulnerable to hydrolytic degradation or may form non-functional-lipid–mRNA aggregates that are no longer capable of supporting protein translation [29]. These findings are consistent with prior reports indicating that high-temperature stress disrupts nanoparticle architecture and compromises RNA integrity [30].

To mimic temperature fluctuations encountered during transportation or cold-chain interruptions, we also investigated the effects of repeated freeze–thaw (F/T) cycles. Surprisingly, luciferase expression was largely preserved following F/T treatment, with no significant reduction in protein output up to a 1.6 ng/μL mRNA concentration. At the highest concentration tested (3.2 ng/μL), an unexpected increase in luciferase activity was observed. Despite previous published AUC data [29] indicating partial nanoparticle destabilization and mRNA leakage post-F/T, the formulation remained functionally active, suggesting that a portion of the mRNA cargo remained both intact and translationally competent.

### 2.4. Effect of ApoE Addition on LNP Encapsulation and Structure

As has been previously described [22], LNP uptake and biodistribution are highly dependent on physicochemical parameters such as particle size, surface charge, apparent pKa, and lipid composition [31,32]. Moreover, the protein corona formed by serum components—including albumin and apolipoproteins—can substantially alter nanoparticle–cell interactions [33].

In particular, apolipoprotein E (ApoE) plays a pivotal role in facilitating LNP cellular uptake and its incorporation into lipid nanoparticle (LNP) formulations potentially reduce the required LNP dose for effective intracellular delivery. ApoE facilitates this process by binding to low-density lipoprotein receptors (LDLRs) on cell surfaces, promoting receptor-mediated endocytosis of the LNPs [34]. ApoE binds to the cholesterol component of LNPs, promoting targeted uptake in hepatocytes and other cell types expressing LDLRs [33,35]. The incorporation of ApoE into LNP formulations has been shown to enhance transfection efficiency, although its effects can vary depending on the cell line and expression of LDLR family receptors [34].

However, the method of ApoE incorporation—whether during or after LNP formulation—can significantly influence the physicochemical properties of the nanoparticles. Studies have demonstrated that ApoE binding can induce structural rearrangements within LNPs, affecting mRNA encapsulation efficiency (EE) and potentially leading to premature release of the mRNA cargo [22].

To investigate these effects, we evaluated the impact of ApoE incorporation at different stages of LNP formulation on the yield of mRNA encapsulation and nanoparticle characteristics. When ApoE was included during the synthesis process (LNP-4), we observed a marked reduction in the YE of the mRNA cargo (Table 1). This suggests that the presence of ApoE interferes with proper nanoparticle assembly, likely by disrupting lipid–mRNA complex formation or altering the electrostatic balance necessary for efficient encapsulation. These findings are consistent with previous reports showing that ApoE can modify the distribution of mRNA within LNPs even when added post-formulation, resulting in a portion of the mRNA being adsorbed on the particle surface rather than encapsulated in the core [22]. In fact, for sample LNP-4, around 60% of mRNA is unencapsulated or surface-associated.

**Figure 4:**
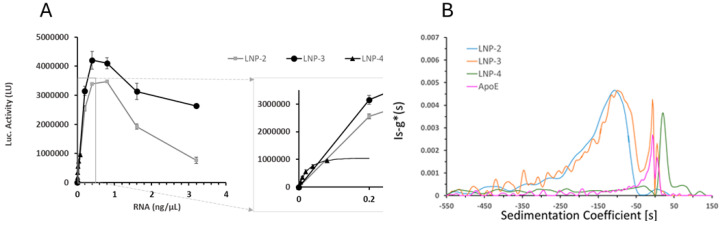
(**A**) Luciferase activity (LU) in A549 cells treated with different LNP formulations: cells were treated with LNP-2 (grey squares), LNP-3 (black circles) at a dose of 0.2, 0.4, 0.8, 1.6, and 3.2 ng/μL, and LNP-4 (black triangles) at a dose of 0.005, 0.01, 0.02, 0.04, and 0.08 ng/μL for 24 h. Values represent the mean ± standard error of the mean for three independent luciferase activity assays. (**B**) Sedimentation coefficient distribution profiles generated with the ls-g*(s) model of Sedfit40 of LNP-2 (blue), LNP-3 (orange), LNP-4 (green), and ApoE protein alone (pink), as determined by analytical ultracentrifugation (AUC). The analysis reveals differences in particle density and integrity among the formulations.

In contrast, when ApoE was added after formulation (LNP-3), the particle size remained comparable to that of LNP-1 and LNP-2 (Table 1), suggesting minimal structural disruption. However, AUC analysis (Figure 4B) revealed a higher particle density—above that of water—unlike standard LNP-mRNA formulations, which typically exhibit buoyant densities below 1 g/mL [36,37]. As protein density is around 1.4 g/mL (>1 g/mL), this shift in density implies a modified nanoparticle architecture and/or altered composition resulting from ApoE binding, potentially due to its interaction with cholesterol moieties on the LNP surface. Figure 4A compares FLuc protein expression of samples with ApoE (LNP-4 and LNP-3) compared to LNP-2.

The results indicate that the addition of ApoE post-LNP-mRNA synthesis (sample LNP-3) increases the amount of functional expressed protein. In the case of ApoE added in the formulation process, LNP-4 is more difficult to assess given that the low yield of encapsulation does not allow a comparable total mRNA dose to be reached in the experiments. The data in the enlarged Figure 4A suggest that LNP-4 can express the encoded protein, but it is premature to make any conclusion on the activity relative to the other samples. These findings underscore the importance of optimizing both the timing and method of incorporating targeting ligands to preserve nanoparticle integrity and ensure functional delivery of mRNA.

## 3. Discussion

We have leveraged our experimental findings to define two critical parameters for evaluating the quality of lipid nanoparticle (LNP)-mRNA formulations: encapsulation efficiency (EE), which quantifies the proportion of RNA encapsulated in the final product relative to the total RNA present, and encapsulation yield (YE), which reflects the proportion of RNA successfully encapsulated compared to the RNA initially used in the synthesis process. As has been demonstrated previously [24], EE can significantly exceed YE. This discrepancy likely arises from incomplete RNA incorporation during LNP assembly or losses during downstream processing steps such as dialysis and filter sterilization. When the formulation process is optimized, EE and YE values converge, but divergence between these metrics in other cases underscores the necessity of distinguishing them explicitly to bring more clarity in the field regarding the description of key properties of the LNP-RNA formulation.

The distinct transfection outcomes observed in the two cell lines highlight the profound impact of cellular context on in vitro gene expression. Variability in post-LNP-mRNA protein expression can stem from inherent cellular differences—such as receptor expression levels, endosomal trafficking dynamics, and translational/excretory capacity—as well as LNP-specific properties, including surface functionality [26,27] and the ionizable lipid composition, which directly influence cellular uptake and endosomal escape efficiency [28]. The striking disparity in luciferase expression between the two cell lines using the same LNP-1 formulation underscores the critical need to select appropriate cellular models for in vitro potency assays. Furthermore, the total treatment volume directly affects the absolute protein yield (Figure 2), emphasizing that experimental conditions—particularly the mRNA dose per well—must be rigorously reported and standardized to ensure reproducibility. Our findings advocate for reporting and comparing mRNA delivery efficacy based on the total mRNA mass per well, rather than concentration alone, to enhance cross-experiment and cross-laboratory comparability.

A notable observation was the retention of functional activity in LNP-mRNA formulations after freeze/thaw cycles. This observation aligns with prior studies demonstrating that minor nanoparticle damage does not always compromise delivery efficiency, provided that residual particles remain intact and functional [38]. This highlights the limitations of relying solely on physicochemical metrics (e.g., size, zeta potential) for quality control. Functional assays—such as cell-based protein expression analyses—are indispensable for capturing true biological activity and should be integrated into standard characterization and quality assurance protocols.

Collectively, our results emphasize the pivotal role of cell-line selection, storage stability, and targeted delivery mechanisms in determining the transduction efficiency of LNP-mRNA formulations. These insights provide the basis for designing more robust, scalable, and effective RNA-based therapeutics. By delineating key variables and experimental scenarios, this work set the scene for the adoption of standardized testing frameworks to ensure consistency, reproducibility, and meaningful benchmarking across research and industrial settings. Such harmonization could facilitate the development of universally applicable metrics for evaluating LNP-mRNA performance, advancing the field toward more reliable and comparable outcomes.

While we acknowledge that aspects of our study—such as the use of conventional cell lines and luciferase mRNA reporters—rely on well-established methodologies, we emphasize that our work addresses a critical yet often overlooked challenge: the reproducibility of in vitro assays for LNP-mRNA formulations. By systematically identifying parameters that govern the consistency and interpretability of experimental outcomes, we provide a framework to mitigate variability and enhance the reliability of in vitro readouts. The strengths of this study lie in its systematic delineation of critical parameters—encapsulation efficiency (EE) and encapsulation yield (YE)—to clarify ambiguities in LNP-mRNA formulation characterization. By demonstrating how EE and YE diverge due to formulation inefficiencies (e.g., incomplete RNA incorporation or losses during dialysis), the work provides a framework for optimizing LNP production and quality control. Furthermore, the emphasis on functional assays over physicochemical metrics (e.g., size, zeta potential) aligns with prior findings that minor nanoparticle damage does not necessarily compromise biological activity, underscoring the necessity of integrating cell-based protein expression analyses into standard protocols. The study’s focus on reproducibility through standardized reporting of the mRNA mass per well, rather than concentration alone, addresses a critical gap in in vitro assay harmonization.

This is particularly important for developers and regulators seeking robust quality control metrics and standardized formulation comparisons, which are essential for advancing RNA therapeutics from research to clinical application. Furthermore, the clarity and practical focus of our approach offer educational value, serving as a training resource for students and early-career researchers in the field. While these insights are familiar to pharmaceutical companies—given their proprietary nature and the confidential nature of industrial R&D—they have not been widely shared with the broader scientific community. By explicitly articulating the know-how in an open-access format, our work aims to bridge this gap, providing a publicly accessible framework to advance reproducibility, standardization, and cross-sector collaboration in the development of RNA-based therapeutics.

However, limitations must be acknowledged. The reliance on conventional cell lines and luciferase mRNA reporters, while methodologically robust, may not fully capture the complexities of in vivo environments or diverse therapeutic payloads. Additionally, the study’s focus on short-term stability (e.g., freeze/thaw cycles) does not address long-term storage challenges or batch-to-batch variability under industrial-scale production. The observed variability in ApoE incorporation methods (during vs. post-formulation) also highlights the need for further investigation into the interplay between targeting ligands and LNP architecture.

Future work should prioritize expanding the in vitro models to include primary cells and 3D tissue systems to better mimic physiological contexts. Developing universal metrics for LNP-mRNA performance, such as standardized potency assays validated across multiple cell types and expression systems, would enhance cross-study comparability. Additionally, exploring the impact of plasma protein interactions—particularly ApoE’s role in receptor-mediated uptake—on LNP biodistribution and targeting efficiency could advance personalized medicine approaches. Finally, integrating orthogonal analytical techniques (e.g., analytical ultracentrifugation) to assess nanoparticle heterogeneity and stability will strengthen quality assurance protocols.

By addressing these challenges, the field can move closer to harmonized applicable standards for RNA therapeutics, ensuring robust, reproducible, and scalable development pathways from research to clinical application.

## 4. Materials

The lipid mixture was purchased from Precision NanoSystems, Vancouver. The CleanCap^®^ FLuc mRNA modified in the Uridine bases (5-Methoxy-Uridine, 5moU) was purchased from Tebu-Bio (Roskilde, Denmark). Human Recombinant Apolipoprotein E protein was purchased from Abcam (Cambridge, UK).

### 4.1. LNP Formulations

LNPs were synthesized using the Nanoassemblr Ignite^®^ instrument (Cytiva, Chicago, USA) according to the manufacturer’s instructions. Briefly, GenVoy-ILM™ (Cytiva, Marlborough, MA, USA) lipid mixture, composed of ionizable lipid, cholesterol, DSPC, and PEG-lipid in a molar ratio of 50:37.5:10:2.5 in ethanol, was allowed to thaw at room temperature and mixed with CleanCap^®^ FLuc mRNA suspended in an acidic buffer, through the NxGen microfluidic cartridge. The nitrogen-to-phosphate ratio (N/P) between the ionizable lipid and the mRNA was maintained at 4:1, while the flow rate and the aqueous-to-organic ratio were TFR: 12 mL/min and FRR: 3:1 (RNA/lipid), respectively. For all formulations, the initial and final waste volumes were set to 0.45 and 0.05 mL, respectively. After the formulation was processed in the micromixer, ethanol and the acidic buffer were removed by dialysis with a centrifugation step in PBS, at 2000 rpm, 4° C, for ≈2 h using a 10 kDa molecular-weight-cut-off dialysis Amicon^®^ filter (EMD Millipore, Billerica, MA, USA). The formulations were finally sterilized through a 0.22 μm Supor^®^ Membrane Low-Protein-Binding Acrodisc^®^ Syringe Filter and stored at 4 °C until use. LNP-2 was then mixed with a suspension containing apolipoprotein E (ApoE) (1:6 ApoE/mRNA molar ratio) and allowed to equilibrate for 30′ at room temperature under rotation (LNP-3) while LNP-4 was formulated with ApoE already present in the mRNA suspension, during LNP formulation) (1:6 ApoE/mRNA molar ratio).

### 4.2. LNP Characterization

#### 4.2.1. Particle Size, Polydispersity Index

The average hydrodynamic particle size (Z av) and polydispersity index (PDI) of LNPs were measured by dynamic light scattering (DLS), using a Zetasizer Nano ZS90 (Malvern Panalytical Ltd., Malvern, UK) at 25 °C, using a back-scattering angle of 173°, and the following settings: material refractive index of 1.45, absorbance of 0.001, dispersant viscosity of 1.020 cP, refractive index of 1.335, and dielectric constant of 80.4. LNPs were diluted in a proper amount of PBS to reach an attenuation factor of 7 or 8 and each measurement was the average of 11 datasets acquired for 10 s each, with a 30 s delay between measurements.

#### 4.2.2. Quantification of Encapsulation

The mRNA loading in LNP formulations was quantified using a Quant-iT^®^ RiboGreen assay (Thermo Fisher, Waltham, MA, USA). RiboGreen reagent binds RNA, producing a fluorescence signal proportional to the concentration, which is quantified using a standard curve. Since RiboGreen cannot penetrate intact LNPs, encapsulated RNA is quantified indirectly by subtracting the fluorescence of intact LNPs diluted five-fold in 1X Tris-EDTA buffer from that of lysed LNPs diluted five-fold and incubated at 37 °C for 15 min in Tris-EDTA buffer 0.5% (*v*/*v*) Triton X-100 (Sigma Aldrich, UK), a detergent, which disrupts the lipid shell and releases the mRNA. The standard curve was also prepared in 1× TE containing 0.5% (*v*/*v*) Triton X-100 (Sigma Aldrich, UK) to account for any variation in fluorescence. The assay was performed according to the manufacturer’s protocol. Samples were loaded on a black, 96-well plate (Corning, Tewksbury, MA, USA) and analyzed for fluorescence on an Enspire microplate reader (Perkin Elmer, Waltham, MA, USA) at an excitation of 485 nm and emission at 528 nm.

Yield of encapsulation (YE, expressed in %) was then calculated as the proportion of encapsulated RNA relative to the total RNA input used in the formulation process. Instead, encapsulation efficiency (EE %) is calculated as the ratio (in %) between the RNA encapsulated in the LNPs and the total RNA in the final formulation (i.e., after LNP-RNA assembly and post-synthesis processing). It is important to note that YE can be significantly lower than EE, as demonstrated by Schober et al., who compared different EE quantification approaches [39]. In vitro dosing was defined based on the calculated encapsulated dose of FLuc mRNA in the final formulation.

#### 4.2.3. Temperature Stress Conditions

Temperature stresses of +4 °C, +37 °C, and +85 °C were achieved as previously described [29]. Briefly, samples were placed in a thermomixer for 15 min. The freeze and thaw process was achieved by repeating 4 cycles of freezing (−80 °C) and thawing of the sample.

#### 4.2.4. Analytical Ultra Centrifugation

To analyze the sedimentation coefficient distribution of the LNP formulations, sedimentation-velocity-type experiments were performed using a Beckman Coulter Proteonlab XL-I analytical ultracentrifuge (AUC) equipped with an 8-hole rotor. Samples were diluted in PBS (Thermo Fisher Scientific, Waltham, MA, USA) to a final volume of 380 μL loaded in a double sector cell with sapphire windows. ApoE was diluted in PBS to a final concentration of 4.2 μg/μL; PBS was used as a reference. Absorbance at 260 nm was measured to detect RNA presence. AUC was run at 10,000 rpm rotational speed at a nominal temperature of 20 °C.

#### 4.2.5. Calculations and Model Fits

Sedimentation coefficient distributions of the molecules were determined using the Is-g*(s) model of the Sedfit40 program over a range from −550 to +150 Svedberg for the fit, with a linear grid with resolution of 400.

### 4.3. Cell Culture

#### 4.3.1. Cell Maintenance

A549 cells (ATCC^®^ CCL-185™) were maintained in DMEM (Ham) F-12 with Glutamax-I (Cat 31765, Thermo Fisher Scientific, Waltham, MA, USA) containing 10% heat-inactivated fetal bovine serum (FBS) (Cat 7524; Merck, Darmstadt, Germany), penicillin (100 U/mL), streptomycin (100 μg/mL), L-glutamine 2 mM, and 0.5% *v*/*v* Hepes. HEK-293 (ATCC^®^ CRL-1573™) cells were maintained in DMEM, high glucose (Cat 11965, Thermo Fisher Scientific, Waltham, MA, USA) containing 10% heat-inactivated fetal bovine serum (FBS) (Cat 7524; Merck, Darmstadt, Germany), penicillin (100 U/mL), streptomycin (100 μg/mL), and L-glutamine 2 mM. Cells were incubated in a humidified atmosphere containing 5% CO_2_ and 21% O_2_ at 37 °C.

#### 4.3.2. In Vitro Luciferase Expression

The LNP formulations used in this study contain mRNA encoding the firefly luciferase enzyme (FLuc mRNA), thus allowing for the setting up of a cell-culture-based assay using the chemiluminescence emission (catalyzed by the luciferase enzyme) as a read out. Cells were seeded into a 96-well plates at a density of 1 × 10^4^ cells per well and left to attach overnight; then, FLuc mRNA LNPs were used to treat cells at the indicated scalar doses. Luciferase activity was measured 24 h after transfection to assess cell uptake and intracellular translation from the mRNA to functional luciferase by the One-Step^TM^ Luciferase Assay system (BPS-Bioscience, San Diego, CA, USA) according to manufacturer’s protocols. Chemiluminescence emission was read with an EnSpire^®^ Multimode plate reader (Perkin Elmer, Waltham, MA, USA). Luciferase expression was reported as a light unit (LU). Unencapsulated FLuc-mRNA was used as a control.

#### 4.3.3. Cell Viability and MTT Assay

The cytotoxicity of LNPs was evaluated in vitro using the 3-(4,5-dimethylthiazol-2-yl)-2,5-diphenyltetrazolium bromide (MTT, Sigma-Aldrich, Inc., Milan, Italy) colorimetric assay on A549 cells. Cells were plated in 96-well cell culture plates (Corning Inc., Corning, NY, USA) at a density of 5 × 10^4^ cells per well and allowed to adhere for 24 h. Cells were then exposed to the LNPs at increasing mRNA doses (0.2, 0.4, 0.8, 1.6, and 3.2 ng/μL) for 24 h. A negative control (medium) and a positive control (Triton 0.1%) were included. The LNP buffer (PBS) was also assessed and tested at the higher dose (3.2 ng/μL of mRNA). At the end of the exposure time, MTT reagent was added to the cells in fresh complete culture medium at a final concentration of 250 μg/mL. After 4 h of incubation at 37 °C, the supernatant was removed, and the precipitated formazan crystals were dissolved in 200 μL RPMI (Sigma-Aldrich, Inc., Milan, Italy) followed by 50 μL of glycine buffer (0.1 M glycine with 0.1 M NaCl in MilliQ water). The absorbance was measured at 570 nm by the EnSpire^®^ Multimode plate reader (Perkin Elmer, Waltham, MA, USA) using a reference wavelength of 680 nm. Data are expressed as a percentage of mitochondrial activity and reported as the mean ± SD. Three independent experiments were performed, and each condition was run in triplicate.

#### 4.3.4. Statistical Analysis

Data are presented as means ± standard error of at least three independent experiments analyzed in triplicate (Supplementary Materials). Statistical analyses were performed by the unpaired Student’s *t* test (* *p* < 0.05, ** *p* < 0.01, and *** *p* < 0.001). A value of *p*  <  0.05 was conventionally considered to be statistically significant.

## 5. Conclusions

The success of mRNA-based SARS-CoV-2 vaccines has amplified the interest in mRNA therapeutics, paving the way for next-generation treatments targeting infectious diseases, cancer, and rare genetic disorders. Despite this momentum, several challenges must still be solved to fully realize the potential of mRNA-based therapies.

Our results highlight several key considerations for the preclinical development of LNP-mRNA formulations. First, the importance of integrating physicochemical characterization with functional biological assays in the development pipeline must be recognized. Second, robust in vitro cell models are essential for evaluating the activity, toxicity, and comparability of synthetic batches before advancing to more-complex and -costly in vivo animal studies. Third, experimental protocols—including treatment volumes, cell densities, and incubation conditions—must be rigorously developed and harmonized to enable valid comparisons across batches, formulations, and laboratories. Fourth, proper storage conditions and buffer selection are critical to maintaining LNP structural integrity over time, as instability can lead to reduced activity.

Consideration of all the possible experimental variables described in this study will help in designing accurate interlaboratory studies aiming at standardizing in vitro activity measurements of LNP-mRNA formulations. The potency of an LNP-mRNA product is determined by the efficiency of cellular uptake and subsequent expression of the encoded protein—whether it be an antigen for vaccine purposes or an effector molecule for gene therapy. Such an approach not only ensures the reproducibility and reliability of preclinical data but also supports the rational design of safe and effective mRNA therapeutics.

This is also particularly important for developers and regulators seeking robust quality control metrics and standardized formulation comparisons, which are essential for advancing RNA therapeutics from research to clinical application. Furthermore, the clarity and practical focus of our approach offer educational value, serving as a training resource for students and early-career researchers in the field.

## Figures and Tables

**Table 1:** Composition and physicochemical properties of LNP-mRNA formulations.

Sample	Buffer	Cargo	Z av (d, nm)	PDI	Yield of Encapsulation
LNP-1	PBS	Fluc mRNA	69	0.08	95%
LNP-2	PBS	Fluc mRNA	79	0.08	99%
LNP-3	PBS + ApoE	Fluc mRNA	82	0.13	99%
LNP-4	PBS	Fluc mRNA + ApoE	127	0.09	13%

## Data Availability

Dataset available on request from the authors.

## Supplementary Materials

The following supporting information can be downloaded at: https://www.mdpi.com/article/10.3390/ijms26178152/s1.
